# Science, politics, ethics and the pandemic

**DOI:** 10.1136/medethics-2021-107706

**Published:** 2021-07-22

**Authors:** Kenneth Boyd

**Affiliations:** Biomedical Teaching Organisation, Edinburgh University, Edinburgh, Scotland, UK

**Keywords:** ethics, ethics- business, ethics- medical

That they are ‘following the science’ has become the watchword of many politicians during the present pandemic, especially when imposing or prolonging lockdowns or other liberty-restricting regulations. The scientists who advise politicians however are usually careful to add that the decision what to restrict and when is ultimately a political one. In science, as in medical practice, there is a delicate balance to be maintained between confidence in the best available information, and the necessary caveat that the assumptions and calculations on which that information is based are subject to further scientific enquiry. For politicians and the public, moreover, as for patients, whether those informing them are judged to be trustworthy is a necessary consideration, a judgement determined by a variety of personal and political contingencies and circumstances. Ethics, by contrast, unable to appeal to scientific consensus (however revisable) or political authority (however reversible), let alone a confidence-inspiring bedside manner, must rest the case for its essentially contestable assumptions and arguments being judged trustworthy, on its willingness to admit all reasoned voices (including occasionally those that question reason itself) to a conversation that is potentially unending, but in the process often highly enlightening.

That conversation is contributed to in this issue of the Journal by several reasoned voices, mostly on ethical aspects of the COVID-19 pandemic. Relevant to issues on which politicians claim to be ‘following the science’, but also raising fundamental ethical questions, is this month’s feature article. In *Ethics of Selective Restriction of Liberty in a Pandemic,* 1 Cameron and colleagues consider ‘if and when it may be ethically acceptable to impose selective liberty-restricting measures in order to reduce the negative impacts of a pandemic by preventing particularly vulnerable groups [for example, the elderly in COVID-19] of the community from contracting the disease’ [and thereby, for example, increasing the disease burden]. ‘Preventing harm to others when this is least restrictive option’, they argue, ‘fails to adequately accommodate the complexity of the issue or the difficult choices that must be made’. Instead, they propose ‘a dualist consequentialist approach, weighing utility at both a population and individual level’, thereby taking account of ‘two relevant values to be promoted or maximised: well-being and liberty’, as well as the value of equality, ‘protected through the application of an additional proportionality test’. The authors then propose an algorithm to take account of the different values and variables which need to be weighed up. They conclude: ‘Selective restriction of liberty is justified when the problem is grave, the expected utility of the liberty restriction is high and significantly greater than the alternatives and the costs of the liberty restrictions are relatively small both at a population and individual level… Discrimination can be justified under these conditions when it is proportionate and limited to a very specific public health challenge’. The arguments and conclusions of the feature article are discussed in the two Commentaries^2 3^.

In *COVID-19 controlled human infection studies: worries about local community impact and demands for local engagement,* 4 Eyal and Lee review recent arguments which express ‘concern about undue usage of local residents’ direly needed scarce resources at a time of great need and even about their unintended infection’ – and hence a requirement for ‘either avoiding controlled infection trials (CHIs) or engaging local communities before conducting CHIs’. They then examine and compare the evidence of such adverse (and some potentially positive) effects of CHIs with those of conventional field trials and argue that ‘both small and large negative effects on struggling communities are likelier in field trials than in CHIs’. ‘Whether or not local community engagement is necessary for urgent vaccine studies in a pandemic’, they conclude, ‘the case for its engagement is stronger prior to field trials than prior to controlled human infection studies’.

In *Payment of COVID-19 challenge trials: underpayment is a bigger worry than overpayment,* 5 Blumenthal Barby and Ubel consider the impact not on communities but on individuals, and specifically on ‘how much people should be paid for their participation in COVID-19 challenge trials’. Noting recent worries about ‘incentivising people with large amounts of money’, they argue that ‘higher payment that accounts for participant time, and for pains, burdens and willingness to take risks’ constitutes neither ‘undue inducement’ (for which the remedy is strengthening informed consent processes and minimising risks) nor ‘unjust inducement’ of individuals from ‘already disadvantaged groups’: evidence of recruitment to challenge trials worldwide suggests, on the contrary, that participants ‘come from all walks of life’. Nor are these authors convinced that ‘offering substantial payment waters down the altruistic motives of those involved’: ‘altruism and payment’ they argue, ‘frequently coexist. Teachers, physicians, public defenders – they all dedicate their lives to helping people. But few do without compensation.’

In *Money is not everything: experimental evidence that payments do not increase willingness to be vaccinated against COVID-19* 6, Sprengholz and colleagues report on an ‘experiment investigating the impact of payments and the communication of individual and prosocial benefits of high vaccination rates on vaccination intentions.’ In November 2020 over 1,000 ‘individuals from a German non-probabilistic sample’ were asked about their intentions. The ‘results revealed that none of these interventions or their combinations increased willingness to be vaccinated shortly after a vaccine becomes available.’ Given that this experiment was conducted before vaccines became available and only in Germany, the authors suggest that these results ‘should be generalised with caution’, but that ‘decision makers’ also ‘should be cautious about introducing monetary incentives and instead focus on interventions that increase confidence in vaccine safety first’.

In *Voluntary COVID-19 vaccination of children: a social responsibility,* 7 Brusa and Barilan observe a pandemic paradox: ‘while we rely on low quality evidence when harming children by school deprivation and social distancing, we insist on a remarkably high level of safety data to benefit them with vaccination’. The consequent exclusion of children from vaccination, they argue, is unjust and not in ‘the best interest of the child as a holistic value encompassing physical, psychological, social and spiritual well-being’, something which ‘there is no scientific method for evaluating’. Society, rather, ‘has the political responsibility to factor in the overall impact of the pandemic on children’s well-being’ and the ‘ultimate choice is a matter of paediatric informed consent. Moreover, jurisdictions that permit non-participation in established childhood vaccination programmes should also permit choice of vaccines outside of the approved programmes.’ The authors conclude by outlining ‘a prudent and ethical scheme for gradual incorporation of minors in vaccination programmes that includes a rigorous postvaccination monitoring.’

In *Challenging misconceptions about clinical ethics support during COVID-19 and beyond: a legal update and future considerations,* 8 Brierley, Archard and Cave note that the ‘COVID-19 pandemic has highlighted the lack of formal ethics processes in most UK hospitals… at a time of unprecedented need for such support’. Unlike Research Ethics Committees (RECs), Clinical Ethics Committees (CECs) in the UK have neither any ‘well-funded governing authority,’ nor the decision-making capacity over clinical questions which RECs have over research. In 2001 the ‘three central functions of CECs’ were described as ‘education, policy development and case review’: but more recently ‘the role of some was expanding’ and in 2020 the UK General Medical Council ‘mentioned for the first time the value in seeking advice from CECs to resolve disagreements’. Misunderstanding of CEC’s role however began to arise when some courts appeared to ‘perceive CECs as an alternative dispute resolution mechanism’ rather than as providing ‘ethics support, with treatment decisions remaining with the clinical team and those providing their consent.’ The future role of CECs, as well as the nature of patient involvement in them, the authors conclude, will depend on a choice between the ‘flexibility and diversity of the current ethical support system’ and ‘greater standardisation, governance and funding’.

Important ethical issues not directly related to COVID-19 are discussed in this issue’s remaining papers. In *Institutional conflict of interest: attempting to crack the deferiprone mystery,* 9 Schafer identifies, places in historical context, and analyses ethical issues raised by the ‘ mystery’ of why between 2009 and 2015 ‘a third of patients with thalassaemia in Canada’s largest hospital were switched from first-line licensed drugs to regimens of deferiprone, an unlicensed drug of unproven safety and efficacy’. He then considers ‘institutional conflict of interest’ as ‘a possible explanatory hypothesis’.

*The perils of a broad approach to public interest in health data research: a response to Ballantyne and Schaefer* 10 by Grewal and Newson and Ballantyne and Schaefer’s response *In defence of a broad approach to public interest in health data research* 11 debate legal and philosophical aspects of whether ‘public interest’, and how narrowly or broadly this is conceived, is the most appropriate justification of consent waivers for secondary research on health information.

In *Do we really know how many clinical trials are conducted ethically,* 12 Yarborough presents evidence in support of the argument that 'research ethics committee practices need to be strengthed' and then suggests 'initial steps we could take to strengthen them'.

Finally, and returning to how ‘science’ is perceived, in *Lessons from Frankenstein 200 years on: brain organoids, chimaeras and other ‘monsters’* 13, Koplin and Massie make a crucial observation: in ‘bioethical debates, *Frankenstein* is usually evoked as a warning against interfering with the natural order or “playing God”’; but in the novel, Frankenstein’s ‘most serious moral error’ was made ‘not when he decided to pursue his scientific breakthrough (one which might, after all, have helped save lives), but when he failed to consider his moral obligations to the creature he created.’ Today, when, like Frankenstein, ‘modern scientists are creating and manipulating life in unprecedented ways’ such as brain organoids and chimaeras, Koplin and Massie argue, ‘two key insights’ can be drawn from Mary Shelley’s 1818 novel. First, ‘if we have created an entity in order to experiment on it’ we need ‘to extend much consideration to its interests and preferences, not least because ‘scientists cannot always rely on existing regulations to anticipate moral issues associated with the creation of new kinds of organisms’. And second: ‘we should be wary of any prejudice we feel towards beings that look and behave differently from us’ and should ‘interrogate any knee-jerk intuitions we have about the moral status of unfamiliar kinds of beings.’
